# Avian and serpentine endogenous foamy viruses, and new insights into the macroevolutionary history of foamy viruses

**DOI:** 10.1093/ve/vez057

**Published:** 2020-01-12

**Authors:** Pakorn Aiewsakun

**Affiliations:** 1 Department of Microbiology, Faculty of Science, Mahidol University, 272, Rama VI Road, Ratchathewi, Bangkok, 10400, Thailand; 2 Center of Microbial Genomics (CENMIG), Faculty of Science, Mahidol University, 272, Rama VI Road, Ratchathewi, Bangkok, 10400, Thailand

**Keywords:** foamy virus, spumavirus, avian foamy virus, serpentine foamy virus, endogenous foamy virus, endogenous retrovirus, ancient retroviruses, co-evolution, co-speciation

## Abstract

This study reports and characterises two novel distinct lineages of foamy viruses (FVs) in the forms of endogenous retroviruses (ERVs). Several closely related elements were found in the genome of oriental stork (*Ciconia boyciana*) and other was found in the genome of spine-bellied sea snake (*Hydrophis hardwickii*), designated ERV-Spuma.N-Cbo (where 'N' runs from one to thirteen) and ERV-Spuma.1-Hha, respectively. This discovery of avian and serpentine endogenous FVs adds snakes, and perhaps more crucially, birds to the list of currently known hosts of FVs, in addition to mammals, reptiles, amphibians, and fish. This indicates that FVs are, or at least were, capable of infecting all major lineages of vertebrates. Moreover, together with other FVs, phylogenetic analyses showed that both of them are most closely related to mammalian FVs. Further examination revealed that reptilian FVs form a deep paraphyletic group that is basal to mammalian and avian FVs, suggesting that there were multiple ancient FV cross-class transmissions among their hosts. Evolutionary timescales of various FV lineages were estimated in this study, in particular, the timescales of reptilian FVs and that of the clade of mammalian, avian, and serpentine FVs. This was accomplished by using the recently established time-dependent rate phenomenon models, inferred using mainly the knowledge of the co-speciation history between FVs and mammals. It was found that the estimated timescales matched very well with those of reptiles. Combined with the observed phylogenetic patterns, these results suggested that FVs likely co-speciated with ancient reptilian animals, but later jumped to a protomammal and/or a bird, which ultimately gave rise to mammalian and avian FVs. These results contribute to our understanding of FV emergence, specifically the emergence of mammalian and avian FVs, and provide new insights into how FVs co-evolved with their non-mammalian vertebrate hosts in the distant past.

## 1. Introduction

Foamy viruses (FVs) are complex retroviruses, belonging to the subfamily *Spumaretrovirinae*, family *Retroviridae*. Past virus surveillance revealed that they are highly prevalent among mammals, capable of infecting from primates (Switzer et al. 2005; Muniz et al. 2015) to cats (Riggs et al. 1969; Winkler et al. 1997), cows (Malmquist, Van der Maaten, and Boothe 1969; Renshaw and Casey 1994), horses (Tobaly-Tapiero et al. 2000), and bats (Wu et al. 2012). Various efforts of animal genomic analyses led to the discoveries of additional FVs, but in the form of endogenous retroviruses (ERVs), adding xenarthrans (Katzourakis et al. 2009), afrotherians (Katzourakis et al. 2014), reptiles (Aiewsakun, Simmonds, and Katzourakis 2019; Wei et al. 2019), amphibians (Aiewsakun and Katzourakis 2017), lobe-finned fish (Han and Worobey 2012), ray-finned fish (Llorens et al. 2009; Schartl et al. 2013; Ruboyianes and Worobey 2016; Aiewsakun and Katzourakis 2017), and cartilaginous fish (Han 2015; Aiewsakun and Katzourakis 2017) to the list of vertebrates that FVs can infect, or at least could infect in the past. However, the evidence for FV infection in birds is still lacking, which is the last major gap in the FV-host range.

FVs are an important model for the study of macroevolutionary history of retroviruses. Previous studies have shown that they have been broadly co-diversifying with their mammalian hosts since the origin of eutherians, dated back ∼100 million years (myr) ago (mya) (Switzer et al. 2005; Katzourakis et al. 2009, 2014). Together with the wealth of their molecular data, this stable co-speciation history between the two played a crucial role in the establishment of the so-called time-dependent rate phenomenon (TDRP). The TDRP states that the relationship between the number of evolutionary changes estimated by the current bioinformatics tools and their associated timescales could be described very well by a simple power-law function (Aiewsakun and Katzourakis 2015, 2016). Application of the TDRP model to the analysis of FV molecular data suggested that the origin of FVs coincided with, if not preceded, the origin of jawed vertebrates which occurred in the early Palaeozoic Era almost half a billion years ago (Aiewsakun and Katzourakis 2017). Although our knowledge of the ultimate origin of FVs and how they interact with mammals is good, little is known about how they interact with other vertebrates, mainly due to the lack of data.

This study reports and describes for the first time an endogenous avian FV found in the genome of oriental stork (*Ciconia boyciana*). This finding corroborates that FVs can indeed (or at least could) infect all major vertebrate groups. In addition, this study also reports a serpentine FV, again identified as an endogenous FV, found in the genome of spine-bellied sea snake (*Hydrophis hardwickii*). By analysing them together with other FVs and leveraging the TDRP model estimated under the well-established FV-host co-speciation assumption, this study reveals a long-term co-speciation history between FVs and ancient reptilian animals. The results also suggest that mammalian and avian FVs originated from cross-class transmissions ultimately from at least one ancient reptile. Based on the estimated evolutionary timescales, the original reptilian hosts likely belonged to the Toxicofera group, which comprises snakes, lizards, and iguanas as well as their reptilian ancestors. This study provides key insights into the macroevolutionary origin of mammalian and avian FVs, and how FVs interacted with non-mammalian vertebrate hosts.

## 2. Materials and methods

### 2.1 ERV mining and genome annotation

All publicly available whole-genome shotgun sequences of birds and snakes in the National Center for Biotechnology Information (NCBI) database (Supplementary Table S1) were searched for FV-like ERVs by using tBLASTn (Camacho et al. 2009). The envelope (Env) protein of the coelacanth endogenous FV (CoeEFV) was used as an initial query. This was because, unlike the retroviral group-specific antigen (Gag) proteins, for example, it is conserved enough to exhibit detectable pairwise similarity to those of distantly related FV-like ERVs at the amino acid level, but not too conserved such that it would exhibit a high degree of similarity to those of non-FV ERVs, like the retroviral polymerase (Pol) proteins. The analysis returned five contigs from the oriental stork (*Ciconia boyciana*) genome, and one contig from the spine-bellied sea snake (*Hydrophis hardwickii*) genome. They were subsequently used as secondary queries to search for additional FV-like ERVs in the two genomes by using BLASTn (Camacho et al. 2009). This returned eight additional contigs from the oriental stork genome, five of which contained complete solo long-terminal repeats (LTRs). The results are shown in Table 1.

**Table 1.** FV-like ERVs in the genomes of oriental stork (*Ciconia boyciana*; accession number: BDFF02000000) and spine-bellied sea snake (*Hydrophis hardwickii*; accession number: RSAD01000000).

Host genome	Contig accession number	ERV name	Start	End	Length	Genetic element
*Ciconia boyciana*	BDFF02004204	ERV-Spuma.1-Cbo	c628,049	c627,231	818	_Host sequence_|Solo LTR|_Host sequence_
	BDFF02004595	ERV-Spuma.2-Cbo	120,710	121,279 (end)	570	_Host sequence_|Solo LTR|_Host sequence_
	BDFF02004819	ERV-Spuma.3-Cbo	c65,278	c64,686	593	_Host sequence_|Solo LTR|_Host sequence_
	BDFF02004994	ERV-Spuma.4-Cbo	c54,051	c53,492	560	_Host sequence_|Solo LTR|_Host sequence_
	BDFF02011124	ERV-Spuma.5-Cbo	c23,912	c16,052	7,861	_Host sequence_|5′LTR–*gag*–*pol*(pro–RT–RNase–Int)–*env*(LP–SU–TM)–acc–3′LTR|_Host sequence_
	BDFF02014057	ERV-Spuma.6-Cbo	c15,488	c14,875	614	_Host sequence_|Solo LTR|_Host sequence_
	BDFF02022208	ERV-Spuma.7-Cbo	c3,174	c1 (end)	3,174	_RE_|Pol(RNase–Int)–Env(LP–SU)|_end contig_
	BDFF02022209	ERV-Spuma.8-Cbo	1 (start)	7,476 (end)	7,476	_Start contig_|5′LTR–*gag*–*pol*(pro–RT–RNase–Int)–*env*(LP–SU)|_end contig_
	BDFF02030469	ERV-Spuma.9-Cbo	c2,952 (start)	c611	2,342	_Start contig_|5′LTR–*gag*–*pol*(pro–RT)|_RE_
	BDFF02034589	ERV-Spuma.10-Cbo	c3,508 (start)	c1 (end)	3,508	_Start contig_|*env*(SU–TM)–acc–5′LTR|_end contig_
	BDFF02035185	ERV-Spuma.11-Cbo	c2,800 (start)	c1,130	1,671	_Start contig_|*env* (TM)–acc–5′LTR|_Host sequence_
	BDFF02043489	ERV-Spuma.12-Cbo	1 (start)	728 (end)	728	_Start contig_|*env*(TM)|_end contig_
	BDFF02044953	ERV-Spuma.13-Cbo	1 (start)	728 (end)	728	_Start contig_|*env*(TM)|_end contig_
*Hydrophis hardwickii*	RSAD01580453	ERV-Spuma.1-Hha	c5,831 (start)	<c892	>4,940	_RE_|*pol*(Int)–*env*(LP–SU–TM)–acc|_Host sequence_

Genomic elements: LTR, long-terminal repeat; *gag*, *group-specific antigen* gene; *pol*, *polymerase* gene; pro, protease domain; RT, reverse transcriptase domain; RNase, ribonuclease H domain; Int, integrase domain; *env*, *envelope* gene; LP: leader peptide domain; SU: surface domain; TM: transmembrane domain; acc, accessory gene; RE, repetitive element. Cross-out genetic elements were those that were deleted.

**Figure 1.**
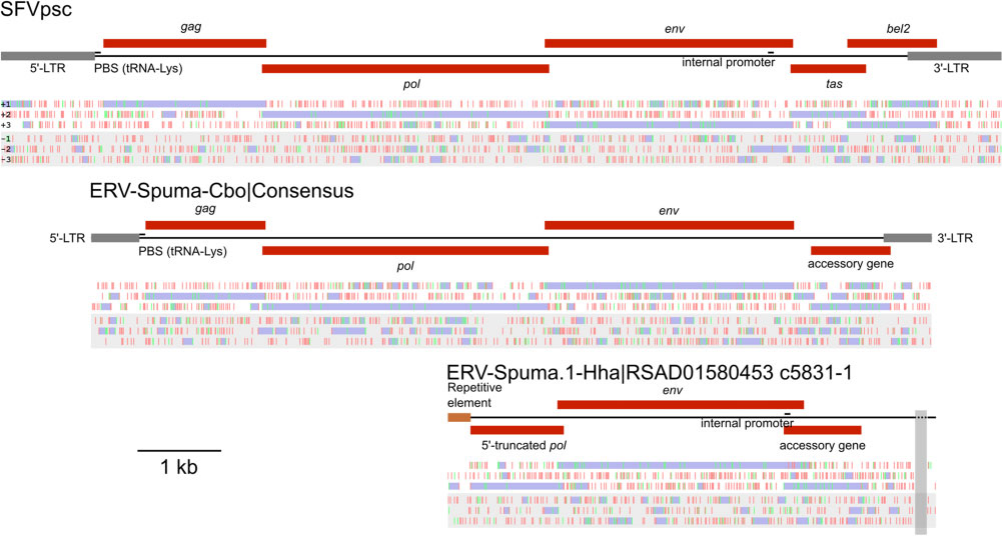
The genomic organisations of simian foamy virus Pan troglodytes schweinfurthii (SFVpsc) (top), consensus ERV-Spuma-Cbo (middle), and ERV-Spuma.1-Hha (bottom). Under each schematic diagram are the distributions of stop (red) and start (green) codons in the six-translation frames (+1, +2, +3, −1, −2, and −3; from top to bottom). Potential open reading frames are shown in purple, and were used to determine potential protein coding regions (red boxes); *gag*: *group-specific antigen* gene; *pol*: *polymerase* gene; *env*: *envelope* gene; *tas*: *transcriptional transactivator* gene; and *bel2*: *bel-*2 gene. Long-terminal repeats (LTRs) are shown in grey. Other identified genomic elements including primer binging sites (PBS) and internal promoters are shown. The vertical transparent grey strip indicates a region of undetermined nucleotide sequences. The scale bar (bottom left) represents a nucleotide length of 1,000 bases.

Manual inspection revealed that all of the thirteen FV-like ERV sequences found in the *C. boyciana* were highly similar, and hence a consensus sequence was estimated for the purpose of genomic characterisation (Fig. 1, Supplementary Fig. S1, and Supplementary Data S1). The consensus sequence of the virus body (Supplementary Data S2) was inferred separately from the LTR portion, and the consensus LTR sequence was inferred from both 5′- and 3′-LTR sequences (Supplementary Data S3). Standard ambiguous bases were used in the case of base count ties. Potential protein coding regions were identified by ORFfinder (https://www.ncbi.nlm.nih.gov/orffinder/). Reciprocal BLASTp searches (Camacho et al. 2009) against the NCBI viral protein database showed that these ERVs exhibited the greatest similarity to modern-day FVs (Table 2), supporting that they were FVs. Thus, the ERVs identified in the genomes of *C. boyciana* and *H. hardwickii* were designated ERV-Spuma.N-Cbo (where ‘N’ runs from one to thirteen) and ERV-Spuma.1-Hha, respectively, according to the recently proposed ERV nomenclature scheme (Gifford et al. 2018). The primer binding sites and internal promoters were located based on sequence homology by using mammalian FVs as references. Figure 1 shows their genomic organisations together with that of simian foamy virus Pan troglodytes schweinfurthii as a mammalian FV reference. The Gag and Env proteins of these ERVs were also used as queries to search for additional FV-like ERVs in other bird or snake genomes, but no significant results were found.

**Table 2.** Reciprocal BLASTp analyses.

Virus	Protein	Best reciprocal BLASTp Hit	Accession number	Query coverage (%)	e-value	% Identity
Consensus ERV- Spuma-Cbo	Gag	gag [Feline foamy virus]	YP_009513248	88	3 × 10^−53^	32.19
	Pol	Pol protein [Yellow-breasted capuchin simian foamy virus]	YP_009508582	98	0.0^a^	47.48
	Env	Envelope protein [Simian foamy virus]	ALJ11212.1	99	0.0^a^	37.29
ERV-Spuma.1-Hha	Pol	Pol protein [*Rhinolophus affinis* foamy virus 1]	AFK85015.1	92	2 × 10^−83^	48.12
	Env	Env [Feline foamy virus]	AGC11914.1	99	0.0^a^	35.84

^a^As explicitly reported by the program.

### 2.2 Phylogenetic analyses

To investigate how the avian and serpentine FVs relate to other FVs, their key retroviral genes, namely the *gag*, *pol*, and *env* genes, were determined and compared (i.e. aligned) against those of other FVs (Supplementary Table S2). The alignments were manually curated to remove regions that could not be aligned confidently. Potential recombination within each alignment was checked by using RDP, GENECONV, Chimaera, MaxChi, BootScan, SiScan, and 3Seq, all implemented in Recombination Detection Program 4 (Martin et al. 2015), with their default settings, but no significant event was found (i.e. those detected by four or more programs).

**Figure 2.**
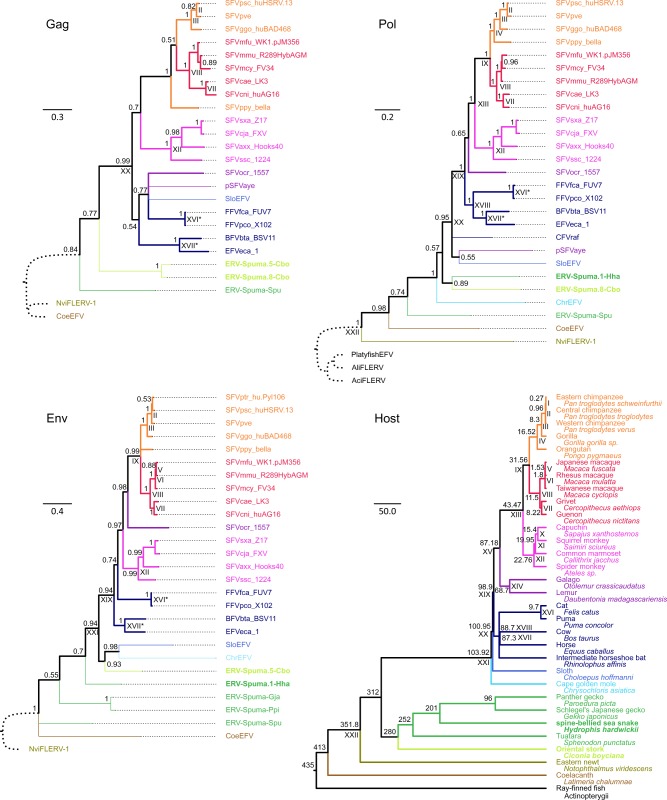
FV phylogenies estimated from the Gag (top left), Pol (top right), and Env (bottom left) proteins, and the phylogeny of the vertebrate hosts (bottom right). FV phylogenies were estimated under the Bayesian phylogenetic framework by using MrBayes 3.2.6 (Ronquist et al. 2012), and were summarised by using the 50 per cent majority rule. Their scale bars are in the units of amino acid substitutions per site. Thin branches are those leading to endogenous FVs, some portion of which may represent neutral evolution. Thick branches are those leading to exogenous FVs, representing pure virus evolution. The outgroups are those with the curve dotted branches. Arabic numerals on nodes are Bayesian posterior probability clade support values. The host phylogeny (see Supplementary Table S2 for virus–host association) was estimated elsewhere (Bininda-Emonds et al. 2007; Stone et al. 2010; Perelman et al. 2011; Jones et al. 2013; dos Reis et al. 2015), and its scale bar is in units of millions of years. Arabic numerals on nodes are diversification dates in units of millions of years, also estimated elsewhere (Supplementary Table S3). Both common and scientific names of the hosts are shown. FV phylogenies were compared with the host phylogeny to identify co-speciation events, labelled with Roman numerals. Only those with ≥75 per cent clade support were considered. Nodes that are labelled with the same Roman numeral are those corresponding to the same co-speciation event. Those labelled with ‘*’ are co-speciation events identified based on topology comparison, but previous studies suggested that they were likely cross-species transmission events. The avian and serpentine FVs reported in this study as well as their hosts are written in bold. Branches and names of the viruses and their hosts are colour coded: orange, apes; red, Old World monkeys; magenta, New World monkeys; purple, prosimians; navy, laurasiatherians; blue, xenarthrans; cyan, afrotherians; green, reptiles; lime, birds; olive, amphibians; brown, lobe-finned fish; and black, ray-finned fish.

Separate phylogenies were estimated from the Gag, Pol, and Env protein sequence alignments under the Bayesian phylogenetic framework implemented in MyBayes 3.2.6 (Ronquist et al. 2012). Two Markov chain Monte Carlo chains were run for 10,000,000 steps, and the parameter values were sampled every 1,000^th^ step with the first 25 per cent discarded as burn-in. The metropolis coupling algorithm (three hot chains and one cold chain) was used to improve the sampling efficiency. Amino acid substitution models used in the phylogenetic reconstruction were the best-fit ones, namely LG+I + Γ(4), RTREV+I + Γ(4), and LG+I + Γ(4) for the Gag, Pol, and Env alignments, respectively, as determined by ModelTest-NG (Darriba et al. 2019) under the Bayesian information criterion. Potential scale reduction factors of all parameters in all analyses were ∼1.000, indicating that all parameter values were well sampled from their posterior distributions and had converged. The phylogenies are shown in Fig. 2.

### 2.3 Evolutionary timescale inference

In this article, evolutionary timescales were estimated by using TDRP models. The TDRP model states that the relationship between the total per lineage substitutions (s estimates) estimated by currently available bioinformatics tools and their associated timescales (t estimates) can be described well by a simple power-law function $t=\alpha s^{\beta }$ (Aiewsakun and Katzourakis 2015, 2016).

Three TDRP models were estimated separately for the Gag, Pol, and Env proteins, mainly based on the co-speciation history between FVs and mammals, which is very well-established (Switzer et al. 2005; Katzourakis et al. 2009, 2014; Ghersi et al. 2015). Indeed, such pattern could be observed in all three phylogenies estimated in this study (Fig. 2). Virus–host co-speciation events were determined by comparing the topologies of the virus phylogenies (Fig. 2, Gag: top left, Pol: top right, and Env: bottom left) against that of the hosts (Fig. 2, bottom right). This provided corresponding s and t estimates required for the TDRP model estimations.

**Figure 3.**
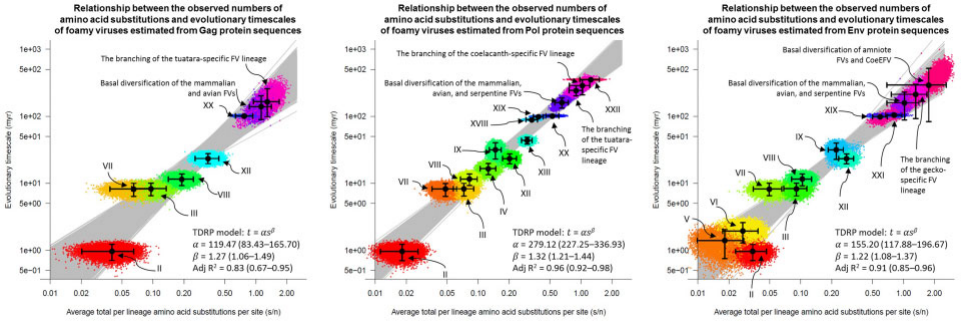
TDRP models estimated from the Gag (left), Pol (middle), and Env (right) proteins. The models were calibrated using average node-to-tip distances (s estimates) and their corresponding evolutionary timescales (t estimates) inferred under the FV-host co-speciation assumption (Switzer et al. 2005; Katzourakis et al. 2009, 2014; Ghersi et al. 2015). Those derived from the co-speciation events are labelled with Roman numerals, referring to nodes in Fig. 2. Solid black dots are median estimates, and the associated 95 per cent HPD intervals are indicated by error bars. A total of 7,500 TDRP models were fitted to the posterior distributions of the s and t estimates (see Materials and Methods). The median model parameter values, adjusted R^2^ scores, and corresponding 95 per cent HPDs (in the parentheses) are shown in the bottom right. The models were extrapolated to infer the dates of various other events as labelled. See Supplementary Table S3 for the values of s and t estimates.

To obtain s estimates, average node-to-tip distances from the inferred co-speciation events to their FV descendants were computed, excluding evolutionary paths leading to ERVs. This was because such paths represented a mixture of both the rapid evolution of exogenous viruses and slow neutral evolution of ERVs, which could result in erroneous models, if included. The timescales of these evolutionary changes (i.e. the t estimates) were inferred directly from those of their hosts, estimated elsewhere (Supplementary Table S3). These corresponding s and t estimates were used to compute the TDRP model parameters; they were first log-transformed and linear models were then fitted to them by using the *lm* function implemented in R (R Core Team 2018). The models were subsequently used to compute the t estimates of other diversification events based on their s estimates (Supplementary Table S3). This procedure was applied to all phylogenies in the posterior distributions obtained from the Bayesian phylogenetic analyses, and the results were used to compute the medians and corresponding 95 per cent highest probability densities (HPDs) for the model parameter values, s estimates, and t estimates (Fig. 3).

## 3. Results

### 3.1 Endogenous FVs in the genomes of oriental stork and spine-bellied sea snake

By querying the NCBI database (Supplementary Table S1) using a series of BLAST searches (Camacho et al. 2009) starting with a CoeEFV Env protein query (see Materials and Methods), thirteen FV-like ERV sequences were retrieved from the oriental stork genome (*Ciconia boyciana*; accession number: BDFF02000000). Five were complete solo LTRs—remnants of retroviral integrations that occur when the two LTRs flanking the proviruses recombine and remove the internal region. The other eights were (parts of) full-length elements containing retroviral genes. The results are summarised in Table 1. The potential retroviral genes were littered with in-frame stop codons, frameshift mutations, and large deletions, as well as were interrupted by transposable elements, indicating that they were *bona fide* ERVs, as opposed to sequences of extant viruses that contaminated the host genomic data.

Examination revealed that all thirteen sequences were highly similar, exhibiting 98.76 per cent nucleotide identity on average in the virus body portion and 96.91 per cent in the LTR portion. This suggested that they were ERVs of the same lineage. The consensus sequence of these ERVs was thus constructed and used for the purpose of genomic characterisation (Fig. 1, Supplementary Fig. S1, and Supplementary Data S1). The alignments used in the consensus sequence construction are available in the supplementary information (virus body: Supplementary Data S2 and LTR portion: Supplementary Data S3). The consensus sequence was 10,043 nucleotide (nt) long. The LTRs were 576 nt in length (5′-LTR: nt 1–576, 3′-LTR: nt 9,468–10,043), relatively short for a FV LTR, which is typically around 950–1,700 nt long (Stoye et al. 2011). A lysine tRNA utilising primer binding site was found downstream of the 5′-LTR (TGGTGCCCAATGTGGGGCTCVA; nt 579–600), commonly utilised by mammalian FVs for reverse transcription initiation (Linial 1999; Lee, Stenbak and Linial 2013). Four potential protein coding regions were identified by examining the distributions of start and stop codons in the six translation frames. Reciprocal BLASTp searches (Camacho et al. 2009) against the NCBI viral protein database suggested that, from the 5′-end, the first three coding regions were *gag* (nt 650–2,083; 1,434 nt long), *pol* (nt 2,046–5,465; 3,420 nt long), and *env* (nt 5,419–8,397; 2,979 nt long) genes, and their protein products were most similar to those of modern-day FVs (Table 2). This finding supported that these ERVs were endogenous FVs, and thus designated ‘ERV-Spuma.N-Cbo’, where ‘N’ is the numeric identifier, running from one to thirteen, according to the nomenclature scheme proposed by Gifford et al. (2018). The hypothetical protein product of the fourth protein coding region (nt 8,601–9,551; 951 nt long) did not exhibit similarity to any known viral or eukaryotic proteins. Given its location however, it was likely an accessory gene, common for a FV.

The BLAST searches also identified one FV-like ERV in the spine-bellied sea snake genome (*Hydrophis hardwickii*; accession number: RSAD01000000) on a relatively short contig (RSAD01580453.1; 5,831 nt long; Fig. 1, Supplementary Fig. S1, and Supplementary Data S4). Three potential protein coding regions were determined. Reciprocal BLASTp (Camacho et al. 2009) searches suggested that the first two from the 5′-end were the 3′ portion of the *pol* gene (RSAD01580453.1: c5,559–4,447; 1,113 nt long), and a full-length *env* gene (RSAD01580453.1: c4,523–1,575; 2,949 nt long). Interrupting the *pol* gene was a repetitive element of an unknown lineage (RSAD01580453.1: c5,831–5,560; 272 nt long), supporting that this was a *bona fide* ERV. Moreover, both protein products showed the greatest similarity to those of modern-day FVs (Table 2). Together, these findings supported that this ERV was an endogenous FV, and thus designated ‘ERV-Spuma.1-Hha’. The protein product of the third potential coding region (RSAD01580453.1: c1,818–892; 925 nt long) did not show similarity to any viral and eukaryotic proteins in the NCBI database. Again, based on its location, it was likely an accessory gene. Unlike in the case of ERV-Spuma.N-Cbo however, an internal promotor (TATAAAA; RSAD01580453.1: c1,809–1,803) could be determined for ERV-Spuma.1-Hha at the expected location towards the 3′-end of the *env* gene by comparing its sequence against those of mammalian FVs. It has been demonstrated that such an internal promotor is required for efficient accessory gene expression in mammalian FVs (Campbell et al. 1994; Löchelt et al. 1995), and hence this might also be the case for the exogenous ancestor of ERV-Spuma.1-Hha.

### 3.2 Phylogenetic analyses

To examine how the avian and serpentine FVs are related to other vertebrate FVs (Supplementary Table S2), their evolutionary histories were estimated from their Gag (Supplementary Data S5), Pol (Supplementary Data S6), and Env (Supplementary Data S7) protein alignments using a Bayesian phylogenetic method (Fig. 2, top left, top right, and bottom left, respectively). ERV-Spuma.5-Cbo and ERV-Spuma.8-Cbo were chosen to represent the avian FVs as they were the longest and the most complete ones (Table 1). Individual phylogenies were estimated from each protein separately to accommodate for their different evolutionary histories as previously noted (Liu et al. 2008; Aiewsakun et al. 2019; Aiewsakun, Simmonds and Katzourakis 2019). Potential recombination was also checked within each alignment (see Materials and Methods). Although various epidemiological and population-level studies have detected recombination within the FV *gag* (Engel et al. 2013; Feeroz et al. 2013), *pol* (Liu et al. 2008), and *env* genes (Winkler et al. 1998; Phung et al. 2001; Galvin et al. 2013; Richard et al. 2015; Aiewsakun et al. 2019), no significant events were found in all of the three alignments used in this study. This was likely due to the fact that they only contained one sequence from each of the host species and/or that different sets of virus sequences were used.

All three phylogenies showed that ERV-Spuma.5/8-Cbo and ERV-Spuma.1-Hha fell within the diversity of known FVs, further supporting that they indeed belonged to the *Spumaretrovirinae* subfamily. Analyses of the Gag, Pol, and Env proteins gave slightly different results, supporting that indeed the three genes have different evolutionary histories (Liu et al. 2008; Aiewsakun et al. 2019; Aiewsakun, Simmonds and Katzourakis 2019), but all three of them showed that ERV-Spuma.5/8-Cbo and ERV-Spuma.1-Hha were most closely related to mammalian FVs. Specifically, the Gag analyses showed that the avian FV proteins were sister to those of mammalian FVs [Bayesian posterior probability (BPP) clade support = 0.77; Fig. 2, bottom right], and not to the tuatara FV (ERV-Spuma-Spu), which is a reptilian FV. This pattern was inconsistent with the host evolutionary history, where birds are more closely related to reptiles than to mammals. The position of ERV-Spuma.1-Hha in the Gag phylogeny could not be determined as its *gag* gene was not found (Table 1 and Fig. 1). In the Pol tree, the avian and serpentine FVs formed a well-supported clade (BPP = 0.89), and appeared to be embedded within the clade of mammalian FVs. The support for the latter pattern was weak however (BPP = 0.57), and thus it could be that the two clades might actually be sisters instead. Nevertheless, it was clear that mammalian, avian, and serpentine FVs grouped together to the exclusion of ERV-Spuma-Spu (BPP = 1.00), again conflicting the host branching orders. Lastly, it was found that the avian FV clustered with mammalian FVs in the Env tree (BPP = 0.94), while the snake FV was basal to the mammalian and avian FV clade (BPP = 0.70).

Regarding other FVs, these analyses produced results that are consistent with previous findings. All three analyses were able to recover that FVs have been broadly co-diversifying with mammals (Switzer et al. 2005; Katzourakis et al. 2009, 2014; Ghersi et al. 2015). The Env analysis showed that the tuatara FV, namely ERV-Spuma-Spu, did not form a clade with ERV-Spuma-Gja, and ERV-Spuma-Ppi, which are gecko FVs, despite the fact that both of them are reptilian FVs, as previously noted (Aiewsakun, Simmonds and Katzourakis 2019). Lastly, both Pol and Env protein analyses showed that amniote FVs were more closely related to CoeEFV, which is a lobe-finned fish FV, than to NviFLERV-1, which is an amphibian FV found in the Eastern newt (*Notophthalmus viridescens*) genome. Again, this phylogenetic pattern conflicted the host evolutionary history, but was consistent with the previous findings (Aiewsakun and Katzourakis 2017; Aiewsakun, Simmonds and Katzourakis 2019). It is noteworthy that, combining all the results together, reptilian FVs appeared to form a paraphyletic group that was basal to mammalian and avian FVs. This phylogenetic pattern strongly suggested that mammalian and avian FVs originated from a series of FV cross-class transmissions ultimately from one or more ancient reptiles.

### 3.3 Evolutionary timescale estimation

To further investigate the deep histories of FVs, their evolutionary timescales were estimated by using TDRP models (see Materials and Methods for details). Such models have been demonstrated to be highly effective at estimating evolutionary timescales of viruses, in particular those of FVs (Aiewsakun and Katzourakis 2015, 2016, 2017; Aiewsakun, Simmonds and Katzourakis 2019; Simmonds, Aiewsakun and Katzourakis 2019).

The models were estimated under the well-established co-speciation assumption between mammalian FVs and their hosts (Switzer et al. 2005; Katzourakis et al. 2009, 2014; Ghersi et al. 2015). Virus–host co-speciation events were determined by comparing the topologies of the virus phylogenies (Fig. 2, Gag: top left, Pol: top right, and Env: bottom left) against that of the hosts (Fig. 2, bottom right). Since the three virus phylogenies were slightly different, different sets of well-supported co-speciation events (those with at least 75 per cent clade support) were inferred (Fig. 2, Gag: 6 events, Pol: 12 events, and Env: 10 events). It was also noted that some co-speciation events obtained from the tree topology comparison alone were likely cross-species transmission events according to the results from previous studies. This included the separations between bovine- and horse-specific FVs (Katzourakis et al. 2014; Aiewsakun and Katzourakis 2015), and cat- and puma-specific FVs (Herchenröder et al. 2019), which had branch lengths that were too short to be consistent with their host evolutionary timescales (Fig. 2, events **XVII** and **XVI** labelled with ‘*’, respectively). These events were thus excluded from the model estimations. Also, based on the tree topology comparison alone, it might be not be immediately obvious that the branching of SFVssc_1224 from other New World monkey FVs (Fig. 2, event **XII**), and the branching of NviFLERV-1 from other amniote FVs in the Pol phylogeny (Fig. 2, event **XXII**) were virus–host co-speciation events. Nevertheless, previous studies have provided temporal evidence strongly suggesting that they both indeed were (Ghersi et al. 2015; Aiewsakun and Katzourakis 2017; Aiewsakun, Simmonds and Katzourakis 2019). They were thus included in the estimations of the TDRP models. In total, three separate TDRP models were estimated for the Gag, Pol, and Env proteins (Fig. 3). These models were subsequently extrapolated to calculate the median estimates and confidence intervals of the timescales of other virus lineages (Fig. 3 and Supplementary Table S3).

Although the Gag, Pol, and Env phylogenies were slightly different in details (Fig. 2), all three of them consistently suggested that the avian and serpentine FVs were most closely related to, and form a clade with, mammalian FVs. The Pol analysis suggested that their most recent common ancestor (MRCA) is ∼159.16 (95 per cent HPD = 132.5–188.81) myr old. Analysis of the Env proteins gave a comparable result, estimating the MRCA to be ∼158.60 myr old, but with a wider uncertainty (95 per cent HPD = 86.02–225.71), reflecting the low clade support (BPP = 0.70). Analysis of the Gag proteins, on the other hand, suggested that their MRCA is ∼138.72 (95 per cent HPD = 90.36–201.61) myr old, which is ∼20 myr lower than those yielded from the Pol and Env analyses.

Regarding the timescales of other FV lineages, the Pol analysis estimated the branching dates of ERV-Spuma-Spu (i.e. the tuatara endogenous FV) and CoeEFV (i.e. the coelacanth endogenous FV) to be ∼242.02 (95 per cent HPD = 202.19–285.18) and ∼288.27 (95 per cent HPD = 211.12–352.94) mya, respectively. Analysis of the Env proteins estimated the gecko FV lineage (i.e. ERV-Spuma-Ppi and ERV-Spuma-Gja) to be ∼212.11 (95 per cent HPD = 84.59–292.43) myr old. Unlike the Pol analysis, the Env analysis could not fully resolve the phylogenetic relationships among CoeEFV, ERV-Spuma-Spu, and amniote FVs (Fig. 2, bottom left); however, the MRCA of this clade was estimated to be ∼292.78 (95 per cent HPD = 80.49–515.30) myr old by the Env protein analysis, comparable to that yielded from the Pol analysis. Basal date estimates obtained from the Env analysis had relatively large confidence intervals (e.g. compared with those from the Pol analysis), and appeared to be largely overlapping with one another (Fig. 3). This could be due to the fact that the uncertainties of the phylogenetic placements of the basal taxa were high (Fig. 2, bottom left). Nevertheless, all of the median age estimates obtained in this study, both from the Pol and Env analyses, were comparable to those previously reported [the age of the tuatara FV linage: ∼232.50–257.15 myr old (Aiewsakun, Simmonds and Katzourakis 2019), gecko FV linage: ∼208.54 myr old (Aiewsakun, Simmonds and Katzourakis 2019), and coelacanth FV linage: ∼262.76 myr old (Aiewsakun and Katzourakis 2017)]. The Gag analysis estimated the tuatara FV lineage to be only ∼164.94 (95 per cent HPD = 97.12–254.05) myr old. This was again considerably younger than that suggested by the Pol analysis [∼242.02 (95 per cent HPD = 202.19–285.18) myr old] and those previously reported [∼232.50–257.15 myr old (Aiewsakun, Simmonds and Katzourakis 2019)], mirroring the results concerning the MRCA of the mammalian, avian, and serpentine FVs.

These systematic anomalies could be due to the small number of data points used to estimate the Gag TDRP model (only six data points, compared with twelve and ten data points for the Pol and Env TDRP models, respectively), which could make it sensitive to the data uncertainties and/or outliers. Indeed, the Gag TDRP model had the lowest goodness-of-fit among all of them [adjusted R^2^: Gag: 0.83 (95 per cent HPD = 0.67–0.95); Pol: 0.96 (95 per cent HPD = 0.92–0.98); Env: 0.91 (95 per cent HPD = 0.85–0.96); Fig. 3]. Consequently, only the timescales obtained from the Pol and Env analyses would be discussed.

## 4. Discussion

Previous efforts of virus surveillance and analyses of animal genomes have led to the discoveries of various distinct lineages of modern and ancient FVs capable of infecting (the ancestors of) mammals (Malmquist, Van der Maaten and Boothe 1969; Riggs et al. 1969; Renshaw and Casey 1994; Winkler et al. 1997; Tobaly-Tapiero et al. 2000; Switzer et al. 2005; Katzourakis et al. 2009, 2014; Wu et al. 2012; Muniz et al. 2015), reptiles (Aiewsakun, Simmonds and Katzourakis 2019; Wei et al. 2019), amphibians (Aiewsakun and Katzourakis 2017), lobe-finned fish (Han and Worobey 2012), ray-finned fish (Ruboyianes and Worobey 2016; Aiewsakun and Katzourakis 2017), and cartilaginous fish (Han 2015; Aiewsakun and Katzourakis 2017). This study reports for the first time avian and serpentine endogenous FVs, found in the genomes of oriental stork (*Ciconia boyciana*) and spine-bellied sea snake (*Hydrophis hardwickii*), designated ERV-Spuma.N-Cbo (where ‘N’ runs from one to thirteen) and ERV-Spuma.1-Hha, respectively (Table 1). These discoveries concluded that FVs are, or at least were, able to infect all five major groups of vertebrates, including mammals, reptiles, birds, amphibian, and fish. Together with other FVs, the deep co-evolutionary history between FVs and vertebrates was investigated in detail, improving our knowledge of FV macroevolution and origin.

Thirteen genomic contigs of *Ciconia boyciana* were found to harbour sequences of avian endogenous FVs, five of which were solo LTRs (Table 1). Examination of their consensus sequence (Fig. 1) revealed that it has a typical structure for an endogenous FV, possessing the three retroviral core genes, namely the *gag*, *pol*, and *env* genes, followed by one accessory gene, and the entire element is flanked by 5′- and 3′-LTRs (Fig. 1). In contrast, only one element of the serpentine FV was found on a relatively small contig of the *Hydrophis hardwickii* genome, designated ERV-Spuma.1-Hha (Table 1). The contig only contained the 3′ half portion of the *pol* gene, a full-length *env* gene, and a long open reading frame, which was likely an accessory gene (Fig. 1). Very remarkably, unlike in the case of ERV-Spuma.N-Cbo elements, the *env* gene of ERV-Spuma.1-Hha had only a few in-frame stop codons. The potential accessory gene also appeared to be fully coding competent, with a potential promoter found inside the *env* gene at the expected location. These findings are consistent with two evolutionary scenarios. One is that ERV-Spuma.1-Hha is young and has not had time to accumulate many mutations yet. An alternative scenario is that its *env* and accessory genes were co-opted by the host for potential cellular functions, and have been maintained under the purifying selection pressure ever since. Indeed, retroviral *env* genes have been co-opted numerous times by various vertebrates, including birds, mammals, and reptiles, for various functions from placenta formation to host antiviral defence (Lavialle et al. 2013; Malfavon-Borja and Feschotte 2015; Denner 2016, 2017; Blanco-Melo, Gifford and Bieniasz 2017; Cornelis et al. 2017). A recent study reported co-options of FV *env* genes by geckos (Aiewsakun, Simmonds and Katzourakis 2019), supporting that this is indeed possible. Further experiments are required to distinguish between these two scenarios.

Phylogenetic analyses of the Gag, Pol, and Env proteins all showed that the avian and serpentine FVs were most closely related to mammalian FVs (Fig. 2). Although their phylogenetic relationships could not be fully resolved, analyses suggested that their MRCA is ∼158.60–159.16 myr old, which is much younger than that of their hosts (Supplementary Table S3). Instead, this age estimate appeared to be highly similar to the time to MRCA of snakes, lizards, and iguanas, also known as the Toxicofera group, estimated to be ∼167 (155–179) myr old (Kumar et al. 2017). This observation strongly supported that the mammalian and avian FVs likely originated from at least one FV cross-class transmission from a member of the Toxicofera group.

Positioned immediately basal to the clade of mammalian, avian, and serpentine FVs were the gecko FV lineage, and subsequently the tuatara FV lineage (Fig. 2). Analyses estimated the ancestor of gecko FVs to branch out ∼212.11 mya, comparable to the one previously reported, ∼208.54 mya (Aiewsakun, Simmonds and Katzourakis 2019). Remarkably, these dates matched very well with the age of the MRCA of geckos and the Toxicofera group, estimated to be ∼201 myr old (Kumar et al. 2017). Furthermore, this study estimated the branching date of the tuatara FV lineage to be ∼242.02 mya. Again, this was comparable to the ones previously reported [∼232.50–257.15 myr old (Aiewsakun, Simmonds and Katzourakis 2019)], and coincided with the split of the tuatara from other reptiles, dated back ∼252 mya (Kumar et al. 2017). Lastly, the mammalian, avian, and reptilian FVs were found to be closer to the CoeEFV, a lobe-finned fish FV, than to the NviFLERV-1, an amphibian FV, conflicting the branching pattern of their hosts (Fig. 2, bottom right). This study estimated the MRCA of amniote FVs and CoeEFV to be ∼288.27–292.78 myr old, comparable to the previous estimate of ∼267 myr old (Aiewsakun and Katzourakis 2017), but significantly lower than that of their hosts, ∼413 myr old (Kumar et al. 2017).

**Figure 4.**
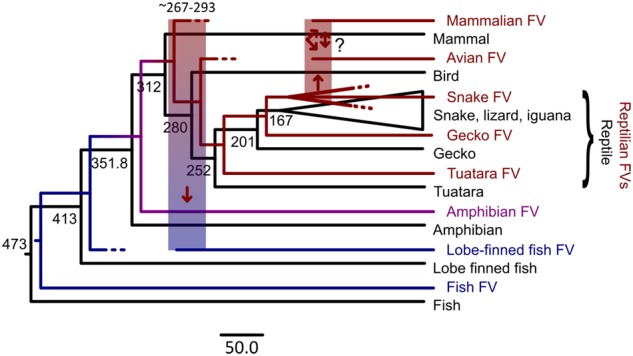
Co-evolutionary history of FVs and their vertebrate hosts. FV phylogeny (coloured) is superimposed onto the host phylogeny (black). The colours indicate if FVs are aquatic (blue), amphibian (purple), or terrestrial (red) FVs. Dotted branches are FV lineages that either have become extinct or have not yet been discovered. Cross-class transmissions are depicted by thick transparent vertical bars and the arrows indicate the direction. The exact transmission route among reptiles, birds, and mammals is unclear (‘?’) due to the limited data availability. The number on nodes are host evolutionary timescales, estimated elsewhere (Kumar et al. 2017) in the units of millions of years. The scale bar is in the units of millions of years.

Based on the results obtained from this study and previous analyses (Aiewsakun and Katzourakis 2017; Aiewsakun, Simmonds and Katzourakis 2019), the macroevolutionary history of FVs could be reconstructed as depicted in Fig. 4. FVs likely originated in the ocean, dated back at least to the origin of the vertebrate hosts almost half a billion years ago. FVs then co-diversified with the early vertebrate hosts into fish, and amphibian FVs, and subsequently reptilian FVs, radiating to the dry land in the process. During this time period, there was one major (chain of) FV cross-class transmission(s) from a land animal back to a sea animal ∼267–293 mya, ultimately giving rise to the ancestor of the CoeEFV found in the coelacanth genome. Based on the estimated timescale and the phylogenetic position of CoeEFV, the ultimate terrestrial animal donor was likely an early amniotic animal.

The results also suggested that the reptilian FVs continued to co-diversify with their hosts into various lineages, including the tuatara-, gecko-, and snake-specific FV lineages. Around the time of, or just after, the basal radiation of the Toxicofera group, there was at least one more major chain of FV cross-class transmissions ultimately from at least one ancient toxicoferan to a protomammal and/or a bird, giving rise to the mammalian and avian FVs that we see today. FVs are typically transmitted through severe bites or scratches involving saliva or blood (Mouinga-Ondémé et al. 2012; Pinto-Santini, Stenbak and Linial 2017), and it is common for reptiles to prey on small mammals and birds. These facts offer a plausible mechanistic explanation for the proposed evolutionary model. Although the exact transmission route among these animals is still unclear, the results suggested that a total of at least two cross-class transmissions are required to explain the relationship among their FVs, and at least one was a jump from a toxicoferan to another animal. For example, it could be that the bird and the protomammal acquired FVs independently from two separate toxicoferan reptiles, or alternatively it could be that the ancestor of mammals acquired the virus first, and later cross-class transmitted to a bird, or vice versa. Additional avian and reptilian FVs are required to shed more light on the exact nature of the transmission route.

## 5. Conclusion

By integrating various sources of genomic information and incorporating the knowledge of the TDRP into the phylogenetic analyses, the results from this study offered several new key insights into the macroevolutionary history of FVs. In particular, this study revealed that birds and snakes are potential FVs’ hosts, corroborating that they could at least in the past infect all major groups of vertebrates. It also provided both phylogenetic and temporal evidence suggesting that FVs co-speciated with ancient reptilian animals, and later cross-class jumped at least two times to a protomammals and a bird, eventually giving rise to the mammalian and avian FVs. The results presented here also confirmed the previous hypothesis that the ancestor of CoeEFV likely originated from one, or a chain of, cross-class transmissions ultimately from a terrestrial amniotic animal back to a fish in the middle Permian Era (Aiewsakun and Katzourakis 2017). Discovery of additional FVs will undoubtedly continue to refine and improve our knowledge about the complex history of this important group of retroviruses.

## 

Conflict of interest: none declared.

## Supplementary Material

vez057_Supplementary_Data
