# Supplementary material for: Avian and serpentine endogenous foamy viruses, and new insights into the macroevolutionary history of foamy viruses
Source: Virus Evol. 2020 Jan 12;6(1):vez057. doi: 10.1093/ve/vez057 (PMC6955096; doi:10.1093/ve/vez057)
Supplement: vez057_Supplementary_Data [file vez057_supplementary_data.zip › Figure S1 ERV-Spuma-Cbo consensus sequence and ERV-Spuma.1-Hha sequence.pdf]

ERV-Spuma-Cbo|Consensus

5′-LTR

1 **TG**TCGCAGGGCACAGAGTAGCCCTTGGTGGAGTAAGGAGCGRTCTGACCACYGTCAAAGCCTCCCACGAACAAATAACAAATAGAGATAG

91 GTTGCTAAACCGCAAAGGCAGACTCACATATGCTTGCTTGCTTAAGTAGGTTYGAATCCCTGAAAYAGAGGATTTCCTGTTTGAACATCA

181 CTCGGTACATAAACATCGAACAAGTTCTGGTATTATGCTACCTAYGTAAAAGATGAAGGTGAAGGTGTATAAAAGTAAGATCGAGTGTAA

271 AGCCAGCAGATCTCCAGCCCATTGGCACTTGGTTGCATCCAAGCAAGGAGACCTCTGGCTCGGTATGTAAATCTTATTATTTAATGCTTG

361 CTCAGGTCACTTAATATTTTATTATTCATTGTGTCTGATACCTCTGTATGATGTGTATATAGTAATAAATAGTAATTAATATATCTGCTC

451 CTTGTGGCCCTGTAATTTATTGTCAGGGGCTTAATATAAGCAACCTGTGCGTAATCCCAACTATTCCTGATTGGCCCAGGCCAAGTAGTG

PBS (tRNA-Lys)

541 GAATAAGTGATCTACGTGAGGCCTATACCCACGA**CA**AT**TGGTGCCCAATGTGGGGCTCVA**GGAGCACAATATATAACTGGTACCCTTAGA

Gag ORF start

M A H N L Y N L E S F N Q R V Q M L Y Q R P P R

631 ATAAATCCTAGATCTAGGTATGGCTCATAATCTCTATAATTTAGAGAGTTTTAATCAGAGGGTTCAGATGTTGTATCAACGCCCTCCAAG

H G E N I T I R I M N G P W G T G D R Y T R I R L E L Q D Q

721 ACATGGTGAAAATATCACCATACGCATTATGAATGGTCCATGGGGAACTGGTGATAGATATACACGAATAAGGTTAGAATTGCAAGATCA

G G A N L P I P Q W Q H X D G R I E R T E I V I H A N F A E

811 AGGAGGGGCAAATTTGCCTATCCCCCAGTGGCAACACTRGGACGGACGAATAGAAAGAACTGAAATTGTAATTCATGCAAATTTTGCTGA

T L N W L G Q P P D I N T G V D R H G P M A H E P F T P G D

901 AACATTAAATTGGTTAGGACAACCACCGGACATTAATACTGGAGTGGATCGACATGGGCCAATGGCTCATGAACCTTTTACTCCAGGAGA

E I C E G Y L P I T L E E L E Q L N Q P G N L R I E A A L L

991 TGAGATTTGTGAAGGATATTTACCTATAACCTTGGAAGAATTAGAGCAACTAAACCAACCAGGAAATTTGAGAATAGAAGCAGCATTATT

A R L Y S Q Q R T Q I G T N V G T G G A R A P L V L V Q V A

1081 AGCAAGATTATATAGTCAACAAAGGACACAAATAGGGACAAATGTTGGAACGGGAGGAGCTAGAGCTCCATTAGTACTGGTCCAAGTTGC

S L P F S N I R A A V G X T P M D I K K I F S W M A E R I N

1171 ATCTCTCCCCTTTAGTAATATACGAGCAGCAGTAGGACHGACTCCCATGGATATTAAAAAGATATTTTCCTGGATGGCTGAAAGGATTAA

I L E G V L P H M N N A T R R Q V V N S L V P Y Q L S L N E

1261 TATATTAGAGGGGGTATTGCCCCATATGAATAATGCAACCAGACGGCAGGTGGTTAACTCACTAGTTCCCTATCAATTATCATTAAATGA

E E C V S W D Q I I S C L Y T K A H G H I P T A K L G E E L

1351 AGAGGAATGTGTATCTTGGGATCAAATTATATCATGTTTATATACTAAAGCTCATGGACATATTCCTACTGCTAAATTAGGAGAGGAATT

Q R I S S E Q G I K T A F Q L G L A M T N Q N Y G H V W G I

1441 ACAGAGAATAAGCAGTGAACAAGGTATAAAGACAGCGTTCCAATTAGGATTAGCTATGACAAATCAGAATTATGGACATGTGTGGGGAAT

I K N L V P G Q A P L A E I T R R L E A L P S D Q E R I R Q

1531 CATTAAAAATCTAGTTCCCGGTCAGGCTCCCCTAGCAGAAATTACTCGCAGATTAGAAGCGTTACCAAGTGACCAGGAACGCATAAGGCA

F S T I V D T V Y R M L D L D P T G K R T G T S R A S P V N

1621 ATTTTCAACTATAGTGGATACAGTCTATCGAATGTTAGACCTTGATCCTACAGGAAAAAGGACAGGAACATCAAGGGCTAGTCCAGTGAA

P S P S P Q G K S X G K K P F K K I F P K E A P V I P P T F

1711 TCCCTCTCCCTCTCCTCAGGGTAAGAGTAVAGGCAAGAAGCCTTTTAAGAAGATATTTCCAAAAGAGGCTCCAGTTATACCTCCAACATT

K P F K P E E R R Y P A S Q G R T E P T K G G Y N L R P R V

1801 CAAGCCTTTTAAACCAGAGGAGAGAAGGTATCCGGCATCTCAAGGAAGAACAGAACCTACTAAGGGAGGTTATAACCTTAGACCCAGGGT

T P P P R Y G Q W D Q S S G S N C P G P S A P P P P P P K D

1891 TACTCCTCCTCCACGATATGGACAATGGGATCAGTCTTCAGGATCCAATTGCCCTGGGCCTTCTGCCCCCCCGCCCCCCCCCCCCAAGGA

Pol ORF start

X K P G P S G I R K K I N X I N K N R K D N E S S G P D T R

M K V L V Q I Q

1981 TAABAAGCCAGGTCCTTCAGGAATAAGGAAGAAAATTAACAVGATTAATAAAAATAGAAAGGACAATGAAAGTTCTGGTCCAGATACAAG

Gag ORF end

T D N *

G Q I I E A E W D S G S E I M I L P K E V L K G L L P I K X

2071 GACAGATAATTGAGGCAGAATGGGACTCAGGATCAGAAATAATGATATTACCTAAAGAAGTGTTAAAAGGTCTCTTACCTATTAAAADAA

I K L M T I T G E I E V P V F Y T T I I I D G K N R R I E V

2161 TTAAACTAATGACCATTACTGGGGAAATTGAGGTACCTGTATTTTACACAACAATTATAATTGATGGGAAAAACAGAAGGATAGAAGTGG

A E S P D G Q A L I S A K D T P W I G I T R K E I E L T I R

2251 CTGAATCACCTGATGGACAAGCTTTAATTTCAGCCAAAGATACGCCTTGGATAGGTATAACCAGGAAGGAAATAGAATTAACTATTAGAA

I D I E K I Q R E I L K Q T D L S D Q G K K Q L E Q F F D H

2341 TTGACATTGAGAAGATACAGAGAGAAATTCTGAAACAAACHGATTTGTCAGATCAAGGGAAAAAGCAATTGGAACAATTTTTTGATCATT

F A P L W Q K W E N Q V G H R S I P P H N I A T G K I K P K

2431 TTGCCCCCTTGTGGCAGAAATGGGAAAATCAGGTAGGACATAGGTCAATTCCGCCTCATAACATTGCTACAGGGAAAATAAAACCGAAAC

P Q K Q F K I N P Q A I P S I Q I V I N D L L K Q G V L R Q

2521 CACAGAAACAGTTTAAAATAAATCCTCAAGCTATACCGTCTATACAAATAGTTATAAATGACTTACTCAAACAAGGTGTCCTCAGGCAAG

E T S E M N T P V Y P V P K G E G K W R L V L D Y R A V N K

2611 AAACATCTGAAATGAATACACCAGTTTATCCAGTACCAAAAGGAGAGGGAAAGTGGAGATTAGTACTAGATTATAGAGCCGTGAATAAAG

V T P A I A A Q N C X S T G I L M Q L T X K K Y K T T L X L

2701 TAACCCCAGCCATAGCTGCACAAAATTGTSATTCAACTGGTATTTTAATGCAATTGACAYGGAAAAAGTATAAAACAACACTAGASCTCT

S X X F X X H P I T K D S Q W I T A F T W C G K Q H V W T Q

2791 CTAWKGSTTTTTRKRCCCATCCTATCACCAAAGATAGCCAATGGATAACAGCCTTTACTTGGTGTGGCAAACAACATGTCTGGACTCAGC

L P Q G F L N S P A L F S A D V V D L L K E F P D V S V Y V

2881 TGCCACAAGGATTTCTAAACTCACCAGCCCTATTTTCTGCAGATGTGGTGGATTTATTAAAAGAATTCCCAGACGTCTCAGTATATGTGA

N D I Y F S H D T E K E H L K T T R H L H N I E R G R I Y S

2971 ATGATATATACTTCTCACATGATACTGAGAAAGAACATCTGAAAACCACAAGACATTTGCACAATATTGAAAGGGGCCGGATATATAGTT

F I K K S E I G K H E V N F L G F A I T N E G R G L T D E Y

3061 TCATTAAAAAATCTGAAATAGGTAAGCATGAAGTAAACTTTTTGGGCTTTGCGATTACTAATGAGGGAAGAGGTCTAACAGATGAATATA

K E K L L N L Q P P K T L K Q L Q S I L G F L N F A H P F I

3151 AAGAAAAACTACTCAATTTACAGCCCCCAAAGACTCTTAAGCAACTGCAGAGTATTTTAGGATTTTTGAATTTTGCCCATCCTTTTATTT

S N F V E L V K P L H D A I I K A N N N E P F W E Q K Q Q D

3241 CCAATTTTGTAGAATTGGTAAAACCTCTCCATGATGCCATCATTAAGGCAAATAACAATGAACCCTTTTGGGAACAAAAACAACAAGATG

A L D E L I T A I N Q A A L L T E R D P T K P L A V K L H X

3331 CTTTAGATGAATTAATCACAGCAATTAACCAAGCAGCTCTTTTAACTGAAAGAGACCCAACCAAACCCCTAGCAGTAAAGCTACATGYRT

S P E A G Y I R L Y N X X D R X P F Q Y X S I X F K X A E K

3421 CTCCTGAAGCAGGMTAYATMAGGCTATATAATRTGGSRGATAGATYTCCCTTTCAATACAYTTCTATARTATTTAAGKGAGCYGAAAAAA

R F T X T E K L L X V X Q Y A L I K S F D I A Q G Q M I H V

3511 GATTYACTYYRACTGAAAAACTSTTGRYTGTAAYGCAATATGCTCTAATTAAAAGTTTTGACATAGCTCAGGGACAAATGATTCATGTTT

Y S P L R X P E T L Q X H X I P E R K X L S S X W L K W M S

3601 ACTCTCCATTACGATSCCCTGAGACATTACAAAGMCATASYATCCCTGAAAGAAAAGYTTTATCATCAYGATGGCTAAAGTGGATGAGTC

H J E N P Q I X F H Y D E E L P D L X S X X X P S E K Q V X

3691 AYMTAGAGAATCCACAAATCARATTTCATTACGATGAAGAATTACCTGACTTGRCATCTTYACMWRCCCCCAGTGAGAAACAGGTCASGA

I R P L T E Y K X I Y Y J X X X A X T N E H K R Q X R M X X

3781 TCAGGCCATTGACAGAATATAAACRAATATATTACMTCKACWGAAGKGCAAYAACYAATGAGCATAAAAGGCAGGMCAGAATGGRYRYAG

V Q X I F N P X Y Q V L N V W S I P L G Q H Q A X Y A E X A

3871 TACAAGMCATTTTTAATCCAKRCTACCAAGTTYTAAATGTWTGGAGTATTCCATTAGGGCARCATCAAGCACRGTAYGCAGAAGYRGCRG

A L E F A L Q Q I P M D Q X P X L I I T D S D Y V S K X Y N

3961 CACTAGAATTTGCCCTYCAACAAATCCCRATGGACCAAAMCCCAAKATTAATTATTACAGATTCAGACTATGTCTCTAAASRTTATAATT

S X L E F W E S N X F X N A K X K P L H H I S L W K S I S E

4051 CTRAGCTAGAATTTTGGGAATCTAATKGGTTTYRTAATGCAAAARGCAAACCATTGCACCATATCTCATTATGGAAAAGCATTTCTGAAC

```
     L  K  K  I  X  P  W  V  H  V  T  H  E  P  G  H  X  C  I  X  T  S  V  X  X  A  G  N  A  X
4141 TAAARAAAATTAAKCCCTGGGTGCACGTCACCCATGAGCCAGGGCATYGATGCATCRGGACCAGTGTAYRYASAGCTGGAAATGCAGYAG

     A  D  S  L  A  K  K  A  X  X  I  N  R  X  H  T  K  P  T  I  D  T  B  L  X  Q  C  I  N  X
4231 CTGAYTCACTGGCAAAGAARGCCARTAYGATTAAYAGGRWACATACCAAGCCAACAATAGACACYRACCTYGRGCAATGCATTAATKAAC

     X  T  P  N  X  P  G  Y  X  K  N  Y  K  X  H  K  D  D  Q  G  I  Y  W  I  T  K  P  E  G  E
4321 KTACTCCAAACCYACCAGGATATWAMAAAAATTATAAATRTCATAAAGATGACCAGGGAATATATTGGATTACTAAACCTGAGGGGGAAT

     F  Q  I  P  P  X  T  E  X  H  X  X  T  E  R  A  H  S  S  L  X  H  A  H  F  G  R  D  A  T
4411 TTCAAATACCYCCYRCTACTGAASGACATWYGRTAACTGAAAGAGCTCATTCCTCTTTGGKACATGCACATTTTGGAAGAGATGCCACCT

     L  A  V  L  K  R  X  X  W  W  P  Y  M  I  Q  X  V  Q  Q  V  L  Q  X  X  S  X  C  X  T  Y
4501 TAGCAGTTCTGAAAAGAAASYRYTGGTGGCCATACATGATCCAGAMAGTACAGCAAGTTTTGCAGTMTTRCTCTARATGTRTCACATATA

     N  S  A  N  X  A  P  I  P  H  G  K  R  X  I  P  E  S  P  F  D  I  L  F  I  D  Y  I  X  X
4591 ATTCAGCTAATCRGGCACCCATCCCCCATGGTAAAAGAWCTATCCCTGAGTCTCCTTTTGATATATTGTTTATWGATTATATAKGACYAT

     L  P  K  C  P  G  Q  L  X  Y  V  L  V  I  I  D  X  A  X  S  F  V  W  L  Y  P  T  X  G  P
4681 TACCAAAATGTCCTGGCCAAYTGGAKTATGTTCTGGTGATTATAGATGRGGCAASTAGTTTTGTATGGCTGTACCCCACTRCAGGACCAA

     T  A  Q  A  T  V  X  A  X  T  D  F  C  X  T  A  I  P  K  K  I  H  S  D  Q  G  P  A  F  X
4771 CAGCCCAAGCCACAGTGYGAGCCSTGACTGATTTTTGCRAGACTGCTATTCCCAAGAAAATACAYTCRGACCAAGGTCCTGCCTTTAYGG

     A  D  I  X  K  E  F  A  K  K  Y  N  X  Q  W  E  Y  S  T  P  Y  H  P  Q  X  S  G  K  V  E
4861 CAGATATWWGCAAAGAGTTTGCAAAGAAGTAYAATRTACAATGGGAATACAGCACACCCTACCACCCCCAGARTAGTGGGAAGGTTGAGA

     R  A  N  X  E  V  K  A  A  L  T  K  L  X  G  S  X  P  G  X  X  Y  A  Y  I  L  L  V  Q  L
4951 GGGCGAACGKGGAAGTCAAAGCAGCTCTCACAAAGTTAYYGGGAAGTYGYCCTGGCAASTRGTATGCCTACATTCTGCTAGTACAATTAG

     G  X  N  N  R  P  R  X  S  I  K  R  T  P  F  E  L  L  F  G  V  P  M  N  V  E  F  N  L  T
5041 GCTTMAATAAYAGACCTAGAMCATCTATCAAAAGAACACCATTCGAATTACTCTTTGGGGTACCAATGAATGTAGAATTTAATCTTACTT

     S  D  L  S  R  E  E  Q  L  A  L  L  A  E  I  R  H  T  L  A  T  T  S  T  S  X  P  P  X  S
5131 CAGACCTCTCCAGAGAAGAACAATTAGCACTCCTAGCAGAAATACGACATACATTGGCGACTACCTCCACATCCHCGCCACCACBCTCAC

     P  H  S  W  H  P  L  V  G  L  L  V  Q  E  R  V  A  T  R  R  P  L  Q  P  Q  W  K  P  P  T
5221 CTCATTCATGGCATCCTCTGGTTGGCCTTCTCGTCCAGGAGAGGGTAGCTACTCGACGACCACTACAACCTCAGTGGAAACCACCAACTC

     P  I  I  K  V  L  S  D  R  V  V  E  I  V  D  K  K  G  N  L  R  Q  V  S  I  D  N  L  K  V
5311 CAATTATTAAGGTACTATCTGATCGAGTTGTTGAAATTGTGGACAAGAAGGGCAACCTGAGACAAGTATCAATAGATAATTTAAAGGTAA
                                  Env ORF start                       Pol ORF end
                               M  A  X  S  L  D  Q  X  M  A  W  E  R  E  T  R  C  R  K  A  L  N  L  V
     T  P  H  Q  Q  Q  N  G  X  V  I  G  P  X  D  G  V  G  E  G  D  *
5401 CTCCACATCAGCAACAGAATGGCCDTGTCATTGGACCAGBGGATGGCGTGGGAGAGGGAGACTAGATGTAGAAAAGCACTTAACTTAGTA

      P  S  D  K  T  E  Q  A  K  I  L  F  Q  N  Y  I  E  E  L  D  E  K  I  T  W  K  T  R  C  R
5491 CCATCAGACAAGACAGAACAAGCTAAAATACTGTTCCAGAATTATATTGAAGAATTGGATGAAAAAATAACATGGAAAACACGATGCAGA

      Y  X  G  Y  A  A  C  A  T  S  T  R  I  A  M  W  I  I  F  T  L  L  I  L  M  I  M  L  G  V
5581 TATTVGGGCTATGCTGCATGTGCTACTAGTACTAGGATAGCAATGTGGATTATATTTACCTTACTCATATTAATGATTATGTTAGGAGTA

      T  C  T  V  I  F  R  L  Q  W  K  Y  A  I  E  R  Q  G  P  T  I  T  W  N  Q  T  I  H  Q  S
5671 ACCTGTACAGTAATATTTAGGTTACAATGGAAATATGCTATAGAGAGACAAGGCCCTACAATTACTTGGAATCAAACAATACATCAATCA

      P  S  I  H  R  I  R  R  G  L  Y  H  H  P  L  P  V  N  V  T  I  T  G  L  K  Q  G  L  Y  W
5761 CCATCTATTCATAGAATTAGGAGAGGATTATATCATCATCCATTACCGGTAAATGTGACTATTACAGGATTAAAACAGGGACTATACTGG

      E  P  F  P  K  L  I  V  A  K  E  R  V  L  G  I  S  Q  I  L  I  L  D  L  D  H  M  A  E  A
5851 GAACCTTTTCCAAAACTGATAGTGGCCAAGGAGAGGGTGCTAGGCATATCCCAGATATTGATATTAGATTTGGATCATATGGCTGAAGCA

      N  N  L  G  D  P  V  G  K  E  I  L  T  Q  L  L  N  E  E  M  K  Q  L  K  D  I  T  L  S  F
5941 AACAATTTAGGAGATCCTGTTGGAAAAGAGATTCTTACTCAGCTCCTAAATGAGGAAATGAAACAGCTTAAAGATATTACTCTTAGTTTT

      E  I  P  L  D  G  P  Q  T  Q  Q  E  Y  I  Q  K  K  X  Y  H  E  F  A  H  C  Y  W  I  D  Y
6031 GAAATACCTTTAGATGGTCCACAAACTCAGCAAGAGTATATTCAAAAGAAAHGTTACCATGAATTTGCACATTGTTACTGGATTGATTAT

      K  E  Q  R  K  W  P  E  P  Q  V  I  A  D  H  C  P  H  P  G  R  G  Y  P  R  F  A  S  K  D
6121 AAGGAACAACGAAAATGGCCAGAACCCCAAGTAATAGCTGATCATTGTCCACACCCTGGAAGAGGATACCCTCGATTTGCTAGTAAAGAC
```

Y W V E S P F S V T K Q E W E R L Q R L K Q G A R L Q T Y R
6211 TACTGGGTAGAATCTCCATTCTCTGTTACGAAACAGGAATGGGAAAGGCTACAAAGGTTAAAACAGGGAGCAAGGCTACAGACATATAGG

L P G G H E T F T G A L M C M D D A Y H L W W N K N K M T Q
6301 TTACCAGGGGGACATGAGACATTTACTGGAGCACTAATGTGTATGGATGATGCCTACCATTTGTGGTGGAACAAAAATAAAATGACACAA

K E L F L A Y V T Q I K K L I Q D M K T G K L K K D A L L N
6391 AAAGAGCTATTCTTAGCCTATGTCACTCAGATAAAGAAGCTGATTCAAGATATGAAAACAGGGAAATTAAAAAAGGATGCCCTTCTGAAT

D W H D Q G K G K W F K S M D D L S F C R H P E L T V F L N
6481 GATTGGCATGATCAAGGAAAAGGGAAATGGTTCAAATCCATGGATGACCTGTCATTTTGTAGACATCCAGAACTTACAGTATTTCTGAAT

G T Y Y K H S C M E G D C Q M T R A N I T Q I K G C K N L T
6571 GGCACATATTATAAACACTCATGCATGGAGGGTGACTGTCAGATGACTCGGGCAAATATAACACAGATAAAAGGTTGTAAAAACCTAACA

S S H K H P Y A C Q F X X X I Q N A T G E D F L Q L Q Y Y D
6661 AGTTCACATAAACACCCATATGCATGTCAATTTTRCARRKYGATTCAAAATGCAACTGGGGAAGATTTTCTCCAACTACAATATTATGAT

Q Q Y L L Y P K Y S R M E E V S H G V D M G L L L H D H T F
6751 CAGCAATATTTATTATACCCTAAATATAGCAGGATGGAAGAAGTCAGTCATGGAGTAGATATGGGACTCCTTTTACATGATCACACATTT

P G P W C I E A K Q V R R Q N Y S L Y S L Y Q Q C L F H S Q
6841 CCCGGACCCTGGTGCATTGAAGCTAAGCAGGTCCGGCGACAGAATTATTCACTATATTCTCTATACCAGCAATGTTTATTTCACTCACAA

K H P V D D V I S G M K Q R L X V Q K Q E E G Y P C N L T T
6931 AAACATCCAGTGGATGATGTAATTAGTGGAATGAAGCAGAGATTATHTGTACAAAAACAGGAAGAAGGGTATCCGTGCAACTTAACCACA

C Q P V S I L D I S R G Q A I W G T N E T F L N Y T I I D T
7021 TGCCAGCCAGTCTCAATCTTAGACATATCACGAGGACAAGCTATCTGGGGCACAAATGAAACCTTCCTAAATTATACAATAATAGACACM

P K K S K G C H T K R K R S I T N L Q K I Q A A R L I L G S
7111 CCTAAAAAATCAAAAGGCTGCCATACTAAGCGAAAGAGATCTATTACTAATCTACAAAAGATACAAGCAGCCAGATTGATCCTAGGTAGT

S I T K I A K I S D L N D K E L A K G I H L L R D H L I T F
7201 TCCATTACTAAAATTGCCAAAATATCTGATTTAAATGATAAAGAATTAGCAAAAGGGATACATCTGCTACGAGATCACCTTATTACATTT

A E H T I D D X I Q M S Q T I T T V M I H S H I Q N L R I L
7291 GCTGAACATACAATTGATGATRTGATTCAAATGAGTCAAACCATTACCACAGTAATGATACATTCTCATATACAGAATTTAAGAATCCTT

L T E G K V D W N I L N S T W I Q E Q L R V T D E I M T L I
7381 TTAACTGAAGGCAAGGTTGATTGGAACATTTTAAACTCAACCTGGATACAAGAACAATTACGAGTCACAGATGAAATTATGACTTTAATA

R R T A R G L A Y D I Q Q R V D K P E K G V W E I S L Y Y E
7471 AGAAGAACAGCTAGGGGATTAGCTTATGATATACAACAACGGGTTGACAAACCAGAAAAAGGAGTCTGGGAAATCTCCCTATATTATGAA

I V I P R R I Y S T N W K I I N Y G H L V Y T G N X G G R X
7561 ATTGTTATACCACGAAGAATATATTCTACTAATTGGAAAATTATCAATTATGGTCATCTGGTATATACTGGAAATARAGGAGGAAGARTC

W L K H P X T L I T Q G C G E V K Y L E V R E C Y E Q D Y L
7651 TGGTTAAAACATCCCTRTACCCTAATAACCCAAGGGTGTGGKGAAGTAAAATATTTAGAAGTAAGAGAATGCTATGAACAAGATTATCTT

I C D E V I X H E P C G N Q T G S R C P I M V E P I V S P Y
7741 ATTTGTGATGAGGTCATCRAGCATGAGCCATGTGGTAACCAAACTGGATCCAGATGTCCTATAATGGTAGAACCAATTGTYTCCCCATAC

L R X X P L K N G X Y I V X T S L D E C X X P P Y Q P S L I
7831 CTTAGAAYTGRCCCATTGAAGAATGGGARTTACATAGTGAYGACCAGTTTGGATGAGTGTARCRTACCACCRTACCARCCATCTCTGATC

T V N E T V T C Y G Y E F K X P L R I N T V I Q E N I H X P
7921 ACAGTAAATGAGACAGTGACTTGCTATGGATATGAGTTTAAAYCACCCCTTAGAATAAATACAGTAATACAAGAAAATATACATRTGCCA

P L S V S L P H M I G I I A D L R Q I K X E L A S S W D S V
8011 CCACTGTCAGTCAGCTTACCGCATATGATAGGTATTATAGCTGATCTTAGACAAATAAAAATSGAGCTGGCTTCCTCTTGGGACTCAGTT

R D X X E R A N T E L L R I D L X E G X X P Q W L N R L S E
8101 CGTGATRTGAYTGAGAGAGCAAATACTGAACTACTGAGAATAGATTTACRTGAAGGAKATAYACCTCAATGGTTAAATAGACTGTCAGAA

S I A D I W P A A A G A I K G I A N G I K D L T S G N F G T
8191 TCCATTGCAGACATCTGGCCTGCTGCAGCTGGAGCTATCAAGGGCATAGCTAATGGAATCAAGGATCTAACATCTGGAAATTTTGGTACG

A F D L L A Y A K P V I I G V V L L I I L V L V I K L I S W

8281 GCTTTTGATTTGTTAGCTTATGCAAAACCCGTGATAATAGGAGTGGTTTTGTTGATTATACTAGTGCTAGTCATCAAATTAATCAGTTGG

Env ORF end

I A T K R K T E *

8371 ATTGCTACGAAACGTAAAACAGAGTGAGATGATGGAATCACAAGATCAGGCTACGTTAGCAATGTTATGGCTTTATAGACCTCTTACTGT

8461 AAAAAGTATACTTACTCATTATAAAACTATCCTGGTACCAATGCCGAGTTGTGACCAGGAGTCTACTTTTATTTTTAGACACGCTTGTCG

Acc ORF start

M E T E H T T P L L Q S D

8551 TTATTGTGCTTATATCTATCATAGTAAATATCTAATTAAGCVAAGAGAAAATGGAGACAGAGCATACTACACCACTACTCCAAAGCGATA

N S N I X D G I L S V H T L K P D T K I D H G Q Y P V V M Q

8641 ACTCAAACATABGGGATGGAATCCTATCTGTACACACATTAAAACCAGACACCAAAATCGATCATGGACAGTACCCTGTGGTCATGCAAC

X T L T T L K G A P P V W I Q T P A K E L A X I T L S D R T

8731 HGACCCTTACTACTCTCAAAGGTGCACCACCTGTCTGGATACAGACTCCGGCGAAGGAACTAGCACBGATCACACTATCTGACAGGACDA

K I L P N H M G W P I P M E Q P Q H L L Y F Q S L H H W M Y

8821 AGATATTGCCCAACCACATGGGATGGCCAATCCCGATGGAACAGCCACAACATCTCCTATACTTTCAGAGTCTCCATCACTGGATGTACA

S S S S R N W V Q T S I T T H C L N L V F P P L G Q V S X T

8911 GCAGCTCTTCGAGGAACTGGGTGCAGACCTCGATCACCACTCATTGTCTGAATCTGGTATTCCCTCCCCTGGGACAGGTTTCRAWAACTT

X S K N W I L T L T C C N I A S G L T H G H C Y M G L W E S

9001 MTTCRAAGAATTGGATTTTAACGTTGACATGCTGTAACATAGCTAGTGGATTGACACATGGACATTGTTACATGGGTCTTTGGGAATCTG

G N N G T A E G W L F W G X K Q C L Q A Q G T S N L W C H K

9091 GAAACAATGGGACTGCTGAGGGATGGCTGTTCTGGGGTYGGAAACAATGCCTACAAGCACAAGGTACAAGTAATCTTTGGTGCCATAAGC

P L L L P M H C Y D T K F A F V S X G X Y P V E D C N V C M

9181 CATTATTACTACCCATGCATTGTTATGATACTAAATTTGCATTTGTTAGCDATGGGDCCTATCCGGTTGAAGACTGTAATGTATGTATGC

L W Y K S Y N S N F S K T P W D I R Y Q H L A M L R K N G C

9271 TATGGTATAAAAGCTATAACTCAAATTTTAGTAAGACCCCTTGGGATATTAGATATCAACATCTAGCAATGTTAAGAAAAAATGGATGTA

T N D I Q C S C T N C Q K K I A I R M I A K N Q K D N W Q A

9361 CTAATGATATACAGTGCTCTTGTACTAACTGTCAAAAGAAAATTGCTATACGAATGATTGCTAAGAACCAAAAAGATAATTGGCAGGCTG

V T A R R G C R R A Q S S P W W S K E R S D H X Q S L P R T

9451 TGACGGCAAGGAGAGGG**TG**TCGCAGGGCACAGAGTAGCCCTTGGTGGAGTAAGGAGCGRTCTGACCACYGTCAAAGCCTCCCACGAACAA

Acc ORF end

N N K *

9541 ATAACAAATAGAGATAGGTTGCTAAACCGCAAAGGCAGACTCACATATGCTTGCTTGCTTAAGTAGGTTYGAATCCCTGAAAYAGAGGAT

9631 TTCCTGTTTGAACATCACTCGGTACATAAACATCGAACAAGTTCTGGTATTATGCTACCTAYGTAAAAGATGAAGGTGAAGGTGTATAAA

9721 AGTAAGATCGAGTGTAAAGCCAGCAGATCTCCAGCCCATTGGCACTTGGTTGCATCCAAGCAAGGAGACCTCTGGCTCGGTATGTAAATC

9811 TTATTATTTAATGCTTGCTCAGGTCACTTAATATTTTATTATTCATTGTGTCTGATACCTCTGTATGATGTGTATATAGTAATAAATAGT

9901 AATTAATATATCTGCTCCTTGTGGCCCTGTAATTTATTGTCAGGGGCTTAATATAAGCAACCTGTGCGTAATCCCAACTATTCCTGATTG

9991 GCCCAGGCCAAGTAGTGGAATAAGTGATCTACGTGAGGCCTATACCCACGA**CA**

ERV-Spuma-Hha|RSAD01580453.1:c5831-1

```
   1 GTGAGTCAGCCATGCCGCTCCCACCTGGTCTACCTGGGCTGGTTGAATCGGATCGGCTGTTCTCTTCCGGAGTGGCTTCATGTTGGCTTT

  91 CCCCTGCCTGCTCCTTTCTACCGAAGTCATTTCCAGGTAAGTAGTTGTGGTTTCTTACTGGGGCTACTGGTGGTGTTGGTTGGCGGTTTA
                                                   Repetitive element
 181 TTGTTGGAGTTTGTTGTGTTGTTTCTTGTGTTAGTGGAGGGTTTCCCCCTCCCCTTTTATTTGTGTTTGGTTATTATTTTTTAATTAATT
        5'-truncated Pol ORF
       L  R  D  G  N  C  I  I  Y  R  P  E  G  I  K  I  I  V  P  A  S  Q  R  L  A  I  T  K  H
 271 AATTACGGGATGGAAATTGTATAATATATCGTCCTGAAGGTATTAAGATTATAGTACCAGCCTCTCAACGGTTAGCTATAACAAAACATG

     A  H  N  S  I  G  N  I  H  G  G  R  Q  A  T  L  L  R  L  Q  G  K  Y  W  W  P  N  M  K  Q
 361 CTCATAACTCCATTGGAAATATACATGGGGGAAGGCAGGCCACTTTGTTAAGGTTACAAGGGAAATACTGGTGGCCTAATATGAAACAAA

     I  V  S  M  I  I  R  T  C  P  Q  C  Q  Q  T  N  F  N  T  T  Q  A  P  K  E  K  R  R  K  I
 451 TTGTTTCAATGATAATAAGAACATGCCCTCAATGTCAACAAACCAATTTTAACACAACACAAGCTCCAAAAGAAAAACGAAGGAAAATAC

     P  D  T  P  F  F  R  I  Y  I  D  F  I  G  P  L  P  P  S  N  G  K  Q  H  V  L  V  C  V  D
 541 CGGATACACCATTTTTCCGAATATATATTGATTTTATTGGACCTCTGCCTCCTTCAAATGGAAAACAGCATGTTTTGGTATGTGTTGATC

     H  C  T  G  F  V  W  L  Y  P  T  R  S  Q  S  A  E  A  T  V  T  A  L  T  M  L  V  S  L  N
 631 ATTGTACAGGATTTGTATGGTTATATCCCACCAGGTCTCAATCAGCAGAAGCAACGGTGACTGCTCTCACAATGTTGGTATCACTTAATA

     K  P  I  N  I  H  S  D  Q  G  S  G  F  T  S  Q  V  T  Q  Q  W  A  K  T  W  G  I  N  W  E
 721 AACCAATTAACATTCACTCTGACCAGGGGTCTGGCTTTACTTCACAAGTAACCCAGCAATGGGCAAAAACTTGGGGTATTAATTGGGAAT

     F  S  V  P  Y  H  P  Q  S  C  G  K  V  E  R  K  N  A  D  I  K  R  A  L  T  K  L  L  I  G
 811 TTTCTGTGCCTTACCACCCCCAAAGTTGTGGGAAGGTGGAAAGGAAAAATGCTGACATAAAACGAGCACTCACCAAGTTACTGATTGGGA

     K  P  K  R  W  A  T  L  I  P  F  V  Q  Y  H  L  N  N  H  P  G  N  R  E  I  S  A  Y  E  L
 901 AACCTAAGCGATGGGCCACTCTTATTCCTTTTGTACAATATCACTTGAATAACCATCCTGGAAATAGAGAAATCTCTGCTTATGAGTTAT

     L  Y  G  I  K  P  N  T  D  F  T  P  E  T  P  E  L  T  R  T  E  Q  L  L  L  L  A  D  L  R
 991 TATATGGTATAAAACCTAATACGGATTTTACCCCCGAAACTCCTGAACTAACCAGAACTGAACAACTTCTCCTTTTAGCAGACTTACGTT

     S  S  L  A  E  S  T  A  C  E  T  L  P  E  P  R  N  P  Y  L  P  G  L  Y  V  Q  E  R  V  P
1081 CTTCCTTGGCTGAATCTACTGCTTGTGAAACATTACCTGAACCTCGTAATCCTTATTTACCTGGTCTTTATGTCCAGGAGAGGGTACCTA

     R  P  A  A  L  R  P  K  W  K  K  P  V  I  I  R  S  V  D  K  Q  N  R  T  V  V  V  D  I  N
1171 GACCAGCAGCTTTAAGACCTAAATGGAAGAAACCTGTAATAATTAGAAGTGTGGATAAACAAAATAGGACTGTTGTAGTGGACATAAACC
                                                         Env ORF start
                                                       M  A  M  T  L  K  Q  W  I  Y  W  N  T  Q
     P  G  N  T  R  T  L  S  F  D  N  I  R  L  L  Q  H  G  N  D  I  K  A  M  D  I  L  E  H  P
1261 CAGGAAATACGAGAACTTTATCTTTTGACAATATTAGATTACTACAACATGGCAATGACATTAAAGCAATGGATATACTGGAACACCCAG
                             Pol ORF end
      K  K  E  Q  E  M  A  H  L  T  T  D  E  D  F  Y  H  N  Y  A  Q  D  I  L  E  V  D  D  E  L
     E  E  G  T  G  N  G  T  F  D  N  *
1351 AAGAAGGAACAGGAAATGGCACATTTGACAACTGATGAAGACTTTTACCACAATTATGCTCAAGACATACTTGAAGTAGATGATGAACTA

      L  K  G  K  V  K  W  Q  T  Y  C  K  Y  R  L  Y  T  L  C  A  T  T  S  R  I  M  A  W  M  I
1441 CTAAAAGGAAAAGTAAAATGGCAAACTTACTGTAAATATAGATTATATACTTTATGTGCCACTACTTCTAGAATAATGGCATGGATGATA

      F  F  A  F  L  L  M  I  T  G  T  T  T  A  I  C  I  L  K  Y  Q  W  K  M  A  Q  R  T  P  G
1531 TTTTTTGCCTTTCTGTTGATGATTACAGGAACAACTACAGCAATTTGCATTTTAAAATATCAATGGAAAATGGCACAAAGAACTCCTGGA

      T  I  L  M  W  N  Q  N  F  S  Q  H  Y  Q  P  P  I  L  H  N  H  R  A  K  R  A  V  F  H  Q
1621 ACTATATTAATGTGGAATCAAAACTTTTCTCAACATTATCAACCACCTATCTTGCATAATCATAGAGCCAAAAGAGCTGTTTTTCATCAG

      I  L  E  T  E  V  S  I  T  G  L  P  Q  G  L  Y  F  E  P  Y  P  K  P  I  V  E  R  E  K  I
1711 ATTTTAGAAACTGAAGTAAGCATTACTGGCTTACCACAGGGACTTTACTTTGAGCCATATCCAAAACCAATAGTGGAGCGGGAAAAAATA

      L  G  I  A  Q  V  L  V  L  D  E  G  Q  M  A  A  A  G  H  I  T  S  K  E  S  L  H  T  L  A
1801 TTAGGCATAGCTCAAGTATTAGTATTAGATGAAGGACAGATGGCAGCGGCTGGACACATAACTTCTAAGGAATCTCTCCACACCCTAGCT

      E  I  I  E  E  E  M  N  I  L  K  K  N  T  F  E  F  N  L  P  L  E  D  P  Y  T  Q  K  Q  Y
1891 GAAATCATAGAAGAGGAAATGAATATATTAAAAAAGAACACATTCGAGTTCAATTTGCCTCTTGAAGATCCATATACACAAAAACAATAT

      Q  H  H  R  C  Y  Q  E  Y  G  H  C  Y  L  I  D  F  E  E  K  W  Q  W  P  T  K  S  I  I  A
1981 CAACATCATAGGTGTTATCAAGAATATGGACATTGTTACTTAATTGATTTTGAAGAAAAATGGCAATGGCCCACTAAGAGTATAATAGCT
```

D H V P E P G Y M N L I D G L H Y P D H Q L A K Y R Y E G P
2071 GATCATGTACCAGAACCAGGATATATGAACTTGATAGATGGACTTCATTATCCAGATCATCAGTTAGCAAAATATAGATATGAAGGACCT

Q F V A P K N W S I E K E H I D R I Q A Y R I P G G Q K E I
2161 CAGTTTGTAGCCCCTAAAAATTGGAGTATTGAAAAAGAACATATAGATAGAATTCAGGCCTATAGAATACCAGGAGGACAAAAAGAAATA

Q Q I I T C N E E I Y A E W W D M R N S Y K E N Q R L W E E
2251 CAACAGATAATAACTTGTAATGAAGAAATATATGCAGAATGGTGGGATATGAGAAATTCTTATAAAGAAAATCAGAGATTATGGGAAGAA

Q I L S Q I T E K G K I R N R A I P M E W G G K G E K R L F
2341 CAAATTCTATCCCAAATAACAGAAAAAGGAAAAATTAGGAATAGGGCCATTCCTATGGAATGGGGAGGAAAAGGAGAAAAAAGACTTTTT

K E Q G R F K V K D Q P E L T Y F L N S S Y W S N S H Y E G
2431 AAAGAACAAGGGAGATTCAAAGTAAAAGATCAACCAGAGTTAACATATTTTTTAAATAGTTCCTATTGGTCCAACTCTCACTATGAAGGA

D C G Y W R Q N Y T D K C K D I K N S P Y M C G F M W T N Q
2521 GATTGTGGATATTGGAGACAGAATTATACTGACAAATGTAAAGACATAAAGAATTCTCCATATATGTGTGGGTTTATGTGGACTAATCAA

Y H K Q P E T N T S W I Q C N N I D T Y E N C I I H N K W S
2611 TATCATAAACAACCCGAAACTAATACCTCATGGATACAGTGTAATAACATAGATACTTATGAAAACTGTATCATACATAATAAATGGAGC

S E N S K R D L G Y L S A T Q V I W T P V C I K E M K I K K
2701 TCAGAAAATAGTAAAAGAGATCTAGGATATTTGTCAGCTACCCAAGTAATATGGACACCAGTATGCATTAAGGAAATGAAAATAAAGAAG

K V K N I Y S I Y F T C M N E S K T V P V E D V I T Q L H N
2791 AAGGTAAAAAATATATATAGTATATATTTTACATGTATGAATGAGTCAAAAACAGTCCCAGTAGAAGATGTAATAACTCAATTACATAAT

H L I I Q K D R K G L P C E H Y S A Q C R E I P I R E S Q E
2881 CATCTTATAATACAGAAAGATAGAAAAGGCTTACCTTGTGAGCATTACTCAGCTCAGTGTAGGGAAATTCCTATAAGAGAAAGTCAAGAA

S L F T W S S N I T L E S F Q P K T L E K K E N V C K G N K
2971 AGCTTATTTACATGGTCATCAAATATCACATTGGAAAGTTTTCAGCCAAAAACTTTAGAGAAAAAGGAGAATGTATGTAAAGGAAATAAA

V R K T R S L L S I E N D K R F Q K A G F L T S R A I T K V
3061 GTAAGAAAAACTAGATCATTACTATCAATAGAAAATGATAAAAGATTTCAGAAAGCAGGATTTTTAACAAGTAGAGCCATAACAAAAGTA

A K I S D L N D Q N L R K G V Y M L R D Y S V Q A F E A I Y
3151 GCTAAAATAAGTGATCTAAATGATCAAAATTTAAGGAAAGGAGTTTATATGTTAAGAGACTATTCAGTACAAGCCTTTGAGGCCATTTAT

H D I S M L E E N I A L Q H L H I H L S S L R M T L I E G K
3241 CATGATATAAGCATGCTAGAAGAAAATATAGCTTTACAACATCTGCATATACACTTGTCTTCCCTAAGGATGACTTTAATTGAAGGAAAA

V P W E L I N D T D I Q Y Q L K L T D N Q M R M V R K T A K
3331 GTACCTTGGGAATTAATAAATGATACTGACATACAATATCAATTAAAATTGACAGATAATCAGATGAGGATGGTACGGAAAACTGCTAAA

A L P Y H I E Q R A R T K D W H L H I Y Y E I M I P R K I Y
3421 GCCCTACCATATCATATAGAACAAAGAGCTAGGACTAAAGACTGGCATTTACACATATACTATGAAATAATGATACCTAGAAAAATATAT

S R N W I L H N I G H L T Q T G Q K V S K I W V Q Q P Y E Y
3511 TCAAGAAATTGGATACTACATAATATTGGCCACTTAACACAAACAGGACAGAAGGTATCCAAAATATGGGTGCAGCAACCATATGAGTAT

I N Q D C E G I K Y L H L E Q C N H D T Y T V C D E V I E T
3601 ATCAATCAAGATTGTGAAGGGATAAAATATTTACATCTAGAACAATGCAATCATGATACATACACAGTATGTGATGAGGTAATTGAAACT

E P C G N R T G S D C P V Y T K A V Q T P Y I Q I I A M K N
3691 GAACCTTGTGGAAATAGAACGGGAAGTGACTGCCCTGTATATACAAAAGCTGTACAAACTCCCTATATACAAATAATAGCTATGAAGAAT

G S Y I I M S D T S E C N I P K H Q S S L I T V N S T L T C
3781 GGAAGCTATATTATCATGAGTGATACTTCTGAATGTAATATACCAAAACATCAATCATCTTTGATAACTGTTAACTCAACCTTAACATGT

Y G Y T F S P P L K T E E V Q T A D F E V P R I H L L L P H
3871 TATGGGTACACATTCTCTCCACCCCTAAAAACAGAGGAAGTACAGACAGCTGACTTCGAAGTACCAAGAATTCATCTGCTACTTCCACAC

Acc ORF start

L A G V I A H L R N V E I Q L T S T W E S I K D L I A R T E

M G K Y K R P H S Q N R

3961 TTGGCTGGAGTTATTGCCCATTTAAGGAATGTAGAAATACAGCTGACCTCCACATGGGAAAG**TATAAAA**GACCTCATAGCCAGAACAGAA

Internal promoter

D T L A G V * A R N S S F L N G L A A S V * L S V V L G * L

R Y I S W S L S K E L I I P Q W T C R I S L T V S R S W M T

4051 GATACATTAGCTGGAGTCTGAGCAAGGAACTCATCATTCCTCAATGGACTTGCCGCATCAGTTTGACTGTCAGTCGTTCTTGGATGACTT

* P V A K V I V L I I I E W I V T C V H N F G G I Q K M M M
L T C C K S D C F N N H R M D C N M C T Q F W W D T K N D D
4141 TAACCTGTTGCAAAAGTGATTGTTTTAATAATCATAGAATGGATTGTAACATGTGTACACAATTTTGGTGGGATACAAAAAATGATGATG

Env ORF end

N F Y L L W I K *
E F L P F V D K M N E W P I H E C K M L E P S H P A L P T L
4231 AATTTTTACCTTTTGTGGATAAAATGAATGAATGGCCAATACATGAATGTAAAATGCTTGAACCAAGTCATCCTGCATTGCCAACTTTAG

G I I Y S H V F L G T E P P L T P W E N V F L S R M K S V R
4321 GTATAATTTATTCACATGTTTTCTTAGGAACCGAACCACCTCTCACACCATGGGAAAATGTGTTTTTAAGTAGGATGAAAAGTGTTAGAC

P E Y Q P F A F G H L C K L M P Y L H Q A F D S D R G V M P
4411 CAGAATATCAACCTTTCGCTTTTGGTCATTTATGTAAGTTAATGCCATATTTGCATCAAGCATTTGATTCTGATCGAGGAGTAATGCCTC

Q H Y A I S E Y A R Y R F A E Q G M L V P V P S F K Q Q I I
4501 AACATTATGCTATATCTGAATATGCTAGATATAGATTTGCAGAACAAGGAATGTTAGTACCTGTTCCCAGTTTTAAACAACAAATTATAG

D M V K R N D P K P I P P I R T T S V I Q E R V S R T E T P
4591 ATATGGTAAAAAGAAATGATCCTAAGCCAATACCGCCTATTAGAACCACTTCAGTGATCCAGGAGAGGGTGTCGCGGACTGAAACCCCTC

Q S P T T S N R G P P I W T N S R D V R L W V D A N L P T G
4681 AATCACCTACCACCAGTAATCGTGGACCACCTATATGGACTAATAGTCGGGATGTAAGATTATGGGTAGATGCCAACTTACCTACAGGAT

S I L M L P Q Q Y Q I R R Y I S G S L L S I F D T S V S Q Q
4771 CTATCCTGATGCTTCCACAGCAATATCAGATACGACGATATATATCAGGTTCCTTGTTAAGTATATTTGATACTTCCGTATCACAGCAAA

Acc ORF end

T G R S E A S S G S S R K K G L R K W A S F A N K Q *
4861 CAGGAAGAAGTGAGGCATCTTCCGGTTCCTCTAGAAAGAAAGGATTACGCAAATGGGCGTCCTTTGCTAATAAACAATAAAAGAACATGA

4951 ATAAAAACCATAGTATTCAACATATTCCTCTGCTGCTTAATAGCTCACCTGTTATAAAAGCTCACATTTGAGTGATTAGCCATTGCTGGA

5041 GTTCTAAAGGTTCGAGTCTTGGGCAATGAGCTAAACACCAGTATGAGATGCTAAAAAGCACCATGAATTATTAAATCACCGGTAACAGAG

5131 ATCGATATAAACCTCATCCCTTGTGTGTTATAAAAATCACAAGTGCATGGGGGCTATATCTTTTATAAAAATATAACCTGCTAGAATTAT

5221 GCTTATGTGCTATGTAATAGAATATAAGGTGAATGAAAATACAAAACAGGAGCTACTTTCTTGAACTCAGCTGCGACTGGGGAAAGTACT

5311 CCTGCTCTGATTGGTAATGTATGATTTTATGTAAACCTTGCTCCAATATTATCTGATATCTAATAATAGCCATATGTACTTAAAAGATCA

5401 AATATATTTTGAAGTAGTTGTATAAATAAAACTTGATTATATTCAAGGAAAATTGATGTCTATGATTTTCTAAGAAAGTGACCAAAACGT

5491 GGGAACTGAGTATCAAAACTTTAAATTAAATTTTAGGATTTAAAAGTAAGGGAGATACTTAGAAAAATAGATAGGACCGCAANNNNNNNN

5581 NNNNNNNNNNNNNNNNNNNNNNNNNNNNNNNNNNNNNNNNNNNNNNNNNNNNNNNNNNNNNNNNNNNNNNNNNNNNNNNNNNNNNNNNNN

5671 NNNNNNNNNNNNNNNNNNNNNNNNNNNNNNNNNNNNNNNNNNNNNNNNNNNNNNNNNNNNNNNNNNTCAACGTCTAGGAAATGGGGAGTTCGGAC

5761 CTCTTCACAATGGAAAGCCCCCCCGAGACAAAGGAGCCCCCCCAACAATACAGCCTCTTCGCTAAGCCCTC
