# Supplementary material for: Avian and serpentine endogenous foamy viruses, and new insights into the macroevolutionary history of foamy viruses
Source: Virus Evol. 2020 Jan 12;6(1):vez057. doi: 10.1093/ve/vez057 (PMC6955096; doi:10.1093/ve/vez057)
Supplement: vez057_Supplementary_Data [file vez057_supplementary_data.zip › Avian and serpentine EFVs.Supp Legends.docx]

# Supplementary Legends

Figure S1: ERV-Spuma-Cbo consensus sequence and ERV-Spuma.1-Hha sequence

Table S1: Genomes searched for FV-like ERVs

Table S2: Viruses used in this study and their hosts

Table S3: Evolutionary timescales of animals and their FVs

Data S1: ERV-Spuma-Cbo consensus sequence

Data S2: alignment of ERV-Spuma.N-Cbo sequences – internal region portion

Data S3: alignment of ERV-Spuma.N-Cbo sequences – long terminal repeat portion

Data S4: ERV-Spuma.1-Hha sequence

Data S5: Gag protein alignment

Data S6 Pol protein alignment

Data S7: Env protein alignment
